# Species-specific genes under selection characterize the co-evolution of slavemaker and host lifestyles

**DOI:** 10.1186/s12862-017-1078-9

**Published:** 2017-12-04

**Authors:** B. Feldmeyer, D. Elsner, A. Alleman, S. Foitzik

**Affiliations:** 1 Senckenberg Biodiversity and Climate Research Centre (BiK-F), Molecular Ecology, Senckenberganlage 25, 60325 Frankfurt am Main, Germany; 2 Evolutionary Biology and Ecology, University of Freiburg, Hauptstrasse 1, 79104 Freiburg, Germany; 3 Institute of Organismic and Molecular Evolution, Johannes Gutenberg University Mainz, Johannes von Müller Weg 6, 55128 Mainz, Germany

**Keywords:** Positive selection, Social parasites, *Temnothorax*, Co-evolution

## Abstract

**Background:**

The transition to a parasitic lifestyle entails comprehensive changes to the selective regime. In parasites, genes encoding for traits that facilitate host detection, exploitation and transmission should be under selection. Slavemaking ants are social parasites that exploit the altruistic behaviour of their hosts by stealing heterospecific host brood during raids, which afterwards serve as slaves in slavemaker nests. Here we search for evidence of selection in the transcriptomes of three slavemaker species and three closely related hosts. We expected selection on genes underlying recognition and raiding or defense behaviour. Analyses of selective forces in species with a slavemaker or host lifestyle allowed investigation into whether or not repeated instances of slavemaker evolution share the same genetic basis.

To investigate the genetic basis of host-slavemaker co-evolution, we created orthologous clusters from transcriptome sequences of six *Temnothorax* ant species - three slavemakers and three hosts - to identify genes with signatures of selection. We further tested for functional enrichment in selected genes from slavemakers and hosts respectively and investigated which pathways the according genes belong to.

**Results:**

Our phylogenetic analysis, based on more than 5000 ortholog sequences, revealed sister species status for two slavemakers as well as two hosts, contradicting a previous phylogeny based on mtDNA. We identified 309 genes with signs of positive selection on branches leading to slavemakers and 161 leading to hosts. Among these were genes potentially involved in cuticular hydrocarbon synthesis, thus species recognition, and circadian clock functionality possibly explaining the different activity patterns of slavemakers and hosts. There was little overlap of genes with signatures of positive selection among species, which are involved in numerous different functions and different pathways.

**Conclusions:**

We identified different genes, functions and pathways under positive selection in each species. These results point to species-specific adaptations rather than convergent trajectories during the evolution of the slavemaker and host lifestyles suggesting that the evolution of parasitism, even in closely related species, may be achieved in diverse ways.

**Electronic supplementary material:**

The online version of this article (10.1186/s12862-017-1078-9) contains supplementary material, which is available to authorized users.

## Background

Parasitism is one of the most successful modes of life, as measured by how often it evolved and by how many parasitic species presently exist [1]. Parasites are a taxonomically highly diverse group, and range from intragenomic ‘genetic’ parasites, through microparasites (viruses, bacteria and protozoa) and macroparasites (worms, arthropods and even vertebrates) [2], to brood and social parasites [3, 4].

Parasites are ideal biological models for the study of ecological specialization, speciation mechanisms, diversification and co-adaptation [5]. The relationship of hosts and parasites is one of mutual adaptation with parasites trying to dupe the host, whereas hosts adapt to defend themselves [6–12]. Co-evolutionary dynamics are shaped by numerous factors including life history traits [13, 14], epidemiological characteristics [15, 16], population size [17, 18], fluctuating environmental changes [19], the presence of multiple parasites reviewed by [20], and social interactions within the host taxon reviewed by [21, 22].

Signatures of balancing selection are expected on immunity genes, playing a major role in the co-evolution between micro-parasites and their hosts, whereas genes encoding behavioural or morphological traits, important in social parasites and their hosts, should show signs of positive selection [23].

Social parasitism, is a special form of parasitism, where the social behaviour of the host, rather than its physiology, is exploited [3]. Avian brood parasites, such as cuckoos and cowbirds take advantage of the brood care behaviour of other bird species, and thus avoid the costs of parental care [24, 25]. Several avian brood parasites evolved from non-parasitic ancestors, and started out by exploiting the brood care behaviour of their conspecifics [26]. Similarly, ant social parasites, can arise via sympatric speciation from their later host [27]. Such a transition to a parasitic lifestyle should lead to the selection of traits important for a parasitic mode of life, such as host recognition, circumventing the host defence system, and transmission. Indeed, avian brood parasites lost their ability to build nests [28], a social parasitic wasp needs a specific host species to be successful as parasite [29], and many slavemaking ants are unable to even feed themselves and completely rely on the care of their enslaved host workers [30].

We are just starting to understand the genomic basis of the parasite lifestyle as such [31–33 and authors therein], and some first patterns of convergence, gene losses and gains become apparent [33 and authors therein, 34]. First studies on the genetic basis of social parasitism concentrated either on ant inquilines - social parasites, that secondarily lost the worker caste and inquiline queens live within host colonies [35] or the Cape honeybee (*Apis mellifera capensis*), in which workers invade other colonies and reproduce clonally [36]. Identified candidate loci for this form of social parasitism include genes involved in ecdysteroid signalling, juvenile hormone and dopamine biosynthesis, which may regulate worker ovary activation [37]. A study on three workerless inquiline social parasites of *Vollenhovia* ants in comparison to their *Pogonomyrmex* hosts found little evidence for gene loss, damaging mutations, or shifts in selection regimes, suggesting that regulatory changes, rather than sequence differences play a role in the evolution of these workerless social parasites [35]. However, the genomic basis of the slavemaker lifestyle and its’ peculiarities has never been investigated.

Here we explore the evolution of the slavemaker lifestyle in North American *Temnothorax* ants, a taxon in which slavery evolved several times independently [38]. We specifically focus on three slavemaker species *T. americanus, T. duloticus* and *T. pilagens*, and their three closely related host species, *T. longispinosus, T. curvispinosus* and *T. ambiguus* [39, 40]. The dulotic lifestyle of these three slavemakers is characterized by recurrent and destructive slave raids during summer [39]. During these raids, slavemaker worker raiding parties search for and attack host colonies to steal worker brood. Upon their emergence as adult workers in slavemaker nests, the social behaviours of these enslaved host workers will be exploited by the slavemakers, whose workers lost the ability to care for themselves [41]. While host nests on average contain around 50 workers [42], the number of workers in slavemaker nests is much lower with on average approximately five workers [39, 40, 43]. Moreover, slavemaker workers are only active during the raiding season and do not take over normal worker chores such as brood care and foraging [44, 45]. Each slavemaker species exhibits distinct morphological characteristics (e.g. size and colour), and raiding behaviours [41, 44]. *T. americanus -* the most derived parasite in the group in terms of morphology and behaviour - mainly uses a propaganda pheromone to induce panic among hosts, preventing organized evacuation or nest defence [41, 46, 47]. The strategy of *T. pilagens* is quite variable, and may also depend on the aggressiveness of the host colony [40, 48]. In some instances host workers are killed by stinging, while in other cases the raid is seemingly peaceful without any casualties, facilitating the incorporation of even adult host workers into the slavemaker colony [48]. *T. duloticus* is a fierce slavemaker that mostly stings all opponents to death before taking the brood, resulting in the local eradication of host colonies [41, 43, 44, 49]. Each of the three slavemakers can exploit several host species, but has a clearly preferred host. The derived *T. americanus* uses all three *Temnothorax* species, but focusses when possible on *T. longispinosus* [50]. *T. duloticus* occasionally attacks *T. longispinosus* but prefers *T. curvispinosus* [43] and *T. pilagens* prefers *T. ambiguus* over *T. longispinosus* [40, 48].

Co-evolution between the obligate social parasites and their hosts not only leads to adaptations in slavemakers, but also to counter-adaptations in behavioural, chemical and life history traits in host species and populations [11, 47, 51–54]. Host aggression [54], as well as host defence strategies [55] are linked to geographic variation in parasite pressure. It is known that adaptations to similar ecological conditions may lead to the evolution of similar (convergent) phenotypes in non-related species. The degree to which parallelism extents to the molecular level has recently experienced an upsurge of interest [56–60]. Evidence is ambiguous, with some studies pointing to parallelism, and others to species-specific trajectories [59, 61 and authors therein]. Moreover, it becomes clear that the level of organisation plays a major role in detecting convergent evolution, as the degree of parallelism is predicted to increase from the nucleotide level to features of whole organisms [61]. The North American *Temnothorax* system, with six closely related slavemaker and host species is ideal to study the genetic basis of repeated evolution of phenotypic traits involved in host-parasite co-evolution.

The main objective of this study was to investigate the selective forces shaping the host and slavemaker lifestyles, and the organisational level of convergence. The main questions we tried to answer were: Which genes are under positive selection in slavemakers or hosts? Is molecular parallelism involved in the convergent evolution of slavemaker lifestyles? Do we find convergence on the gene, functional or pathway level?

## Methods

Ant colonies were collected over the course of 2 years (2013 and 2014) in New York State, Ohio and Michigan (Additional file 1), and brought back to the lab in Mainz. To induce raiding activity, colonies were moved during the raiding season in August to 25 °C, 14 L:10D light cycle conditions 1 week prior to the onset of the raiding experiment. Raiding arenas (30 × 40 cm plastic boxes with plastered floor) were set up, in which each slavemaker species was allowed to raid colonies of its preferred host species. We waited until slavemaker scouts had returned to their mother nest and recruited additional raiders to infiltrate the host nest, and aggressive encounters between slavemaker and host workers could be observed. This was the time we sampled workers actively engaging in a raid or nest defence respectively. To obtain workers in a somewhat neutral behavioural state, we collected host individuals outside the nest before raids, as well as slavemaker workers outside the raiding season under the same external conditions. Since we were interested in the evolution of slavemaker and host genes in respect to raiding and nest defence behaviour, we obtained transcriptomes of ants engaged in these respective behaviours. Six workers per species and behaviour were pooled for RNA isolation in replicates of six. Libraries were constructed and sequenced paired-end on an Illumina HiSeq 2000 at GENterprise Genomics. Sequences were quality trimmed with *Trimmomatic* v0.32 [62]. De novo assembly of the transcriptomes was conducted using a combination of the CLC bio workbench (Qiagen) and *MIRA* [63] (Additional file 2; for more details see [64]). Contigs were annotated using *BlastX* v.2.2.30 against the non-redundant arthropod database (November 2014). The online tool *ORFpredictor* 2.0.3 [65] was used to predict open reading frames and amino acid sequences for all contigs. The predicted and translated amino acid sequences were used as input for *OrthoMCL* 2.0.9 [66], to build ortholog sequence clusters. In total we obtained 55,521 orthologous protein clusters, out of which 6432 clusters contained at least one sequence per species. These clusters were filtered with an in-house python script (available from GitHub: https://zenodo.org/record/60135#. V9k495h96Uk) based on pairwise Blast similarity scores, which resulted in 5791 clusters with a single sequence per species. After trimming these sequence alignments with *Gblocks 0.91b* [67], 5199 clusters remained for further analyses (NCBI Bio Project GSE95604).

### Phylogenetic analysis

We chose the myrmicine ant *Acromyrmex echinator* to include as outgroup, for which we observed the highest Blast similarity in our contig Blast searches. *A. echinator* sequences were obtained from the “Hymenoptera Genome Database” [68; aech_OGSv3.8_pep.fa]. We inferred orthology between *T. curvispinosus* and *Acromyrmex echinator* applying a local *BLASTn* [69]. The according sequences for each cluster were obtained and aligned with *Mafft 7.0* [70]. The alignments were trimmed using *Gblocks* 0.91b [66] with default settings. All clusters were concatenated into a single alignment and the program *ProtTest* 3.4 [71] was used to calculate the appropriate evolutionary model (JTT + I + G + F)*.* A *Maximum Likelihood* phylogenetic tree with 1000 bootstrap replicates was constructed with *RAxML 8.1.16* [72]. We additionally estimated evolutionary models for each single cluster and constructed the respective *Maximum Likelihood* trees for the *codeml* analyses (see below).

### Tests for positive and relaxed selection

To test for signatures of positive selection the software package *PAML* 4.8 [73] was used to apply the branch-site model A in *codeml* (model = 2, NSsites = 2). *codeml* estimates the nonsynonymous/synonymous substitution ratio (ɷ = dN/dS), where ɷ = 1 indicates neutral evolution, ɷ < 1 purifying selection, and ɷ > 1 indicates positive selection. To test for statistical significance log-likelihood ratios were calculated and FDR corrected for multiple testing [74]. The cluster specific tree topology, as inferred by *RAxML* was used as input for *codeml*. To test for positively selected genes, we coded each single species as foreground branch, and additionally the set of slavemaker branches as well as the host branches respectively.

The online tool *Venny *2.1 (http://bioinfogp.cnb.csic.es/tools/venny/) was used to visualize shared and species-specific genes. To statistically assess to what extent the observed intersection in divergent features among pairs of species would be expected by chance, we applied a randomisation procedure implemented in a custom Python script. We used 10,000 replicates to infer how often an observed intersection of size i of x and y positive draws from a base population of size z was larger or smaller than those from random draws.

### Enrichment analyses

To obtain identifiers suitable for the enrichment tool *DAVID* 6.7 [75], we inferred orthology by applying a *BLASTx* between *T. curvispinosus* contigs and *Drosophila melanogaster* protein sequences *(dmel-all-translation-r5.56.fasta)* obtained from *flybase* (flybase.org). The complete contig set was used as background and the according positively selected genes as test set. Furthermore, to obtain pathway information in form of KO (KEGG Orthology) assignments for the according gene sets, we utilized *KAAS* [76], an automated annotation server. The KEGG Mapper – Reconstruct Pathway tool was used to obtain the associated pathways (http://www.genome.jp/kegg/tool/map_pathway.html).

## Results

### Phylogenetic analysis

**Fig. 1:**
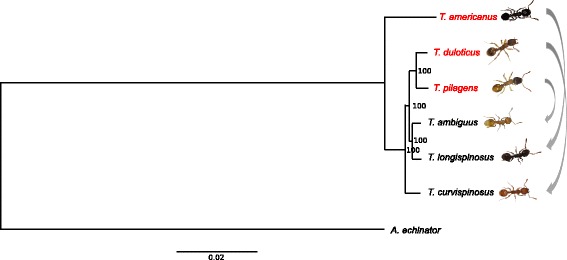
RAxML obtained Maximum Likelihood phylogenetic relationship of the six slavemaker and host species with *Acromyrmex echinatior* as outgroup, and based on 5199 ortholog gene-clusters (ML bootstrap percentages depicted at nodes). Slavemaker species names are given in red, host species names in black. Arrows connect slavemaker-host species pairs

Based on the concatenated sequence of 5199 ortholog sequence clusters the *RAxML* phylogenetic analysis resulted in a tree with well supported nodes (Fig. 1). *T. americanus* is the most distant taxa to the other five *Temnothorax* species, as corroborated by its deviant phenotype and a previous phylogenetic analysis [38]. In contrast to this earlier phylogeny based on two mitochondrial loci [38], our nuclear tree now supports a sister species relationship between the two younger slavemaking species *T. duloticus* and *T. pilagens* [40], as well as between the two host species *T. ambiguus* and *T. longispinosus,* with *T. curvispinosus* being the next distant taxon, followed by *T. americanus*.

### Positively selected genes

**Fig. 2:**
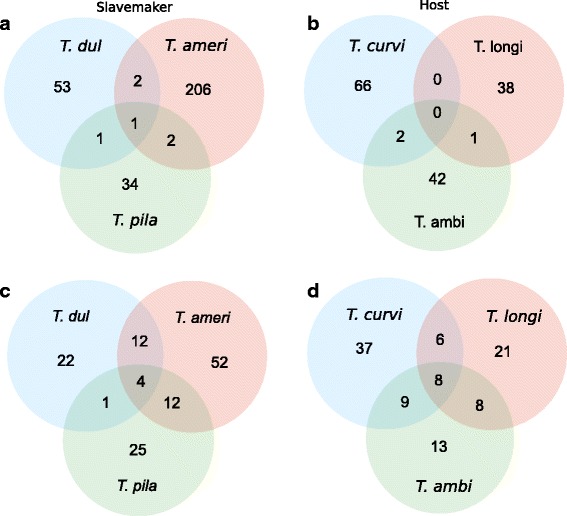
Venn diagrams depicting the number of shared and “private” positive selected genes (**a** + **b**) and corresponding pathways (**c** + **d**) in slavemakers and hosts. (*T. dul = T. duloticus, T. ameri = T. americanus, T. pila = T. pilagens, T. curvi = T. curvispinosus, T. longi = T. longispinosus, T. ambi = T. ambiguus*)

In total, we found 574 genes under positive selection; 309 on the branches leading to slavemakers, and significantly less, 161, positively selected genes on the branches to hosts (χ^2^_1_ = 77.85, *p* < 0.0001; Additional file 3). Looking at the branches of each single species, we detected more than four times as many genes under selection in the derived slavemaker *T. americanus* (*N* = 211) than in the two younger slavemaker sister species *T. pilagens* (*N* = 38) and *T. duloticus* (*N* = 54; Fig. 2a). The host species *T. ambiguus* shares one gene under positive selection with its sister species *T. longispinosus*, and two genes with *T. curvispinosus*, whereas the latter two species do not share any positively selected genes (Fig. 2b). In slavemakers, one positively selected gene (a hypothetical protein) is shared amongst all three species, *T. americanus* shares two genes each with the two other slavemakers, and *T. pilagens* shares only one positively selected gene with its sister *T. duloticus* (Additional file 3). In both, hosts and slavemakers, the number of shared genes under positive selection among species is less than one would expect by chance (Additional file 4: Table S1). Moreover, we compared the positively selected genes to differentially expressed genes from an accompanying gene expression analysis [64] based on the same host and slavemaker transcriptomes. Six genes in hosts and 36 in slavemakers appeared in both, the differential expression and the positive selection analyses (Table 1). None of the positively selected gene sets per species, in slavemakers or hosts, were enriched for any functional category.

**Table 1:** List of genes which emerged as candidates in this study (signatures of positive selection), as well as an accompanying study contrasting gene expression patterns during a raid versus no raid behaviours [64]

*T. ambiguus*	*T. curvispinosus*	*T. longispinosus*
hypothetical protein G5I_08161	Trypsin-7	Leukotriene A-4 hydrolase
hypothetical protein SINV_10379	Suppressor of tumorgenicity protein 14	
Trypsin-7		
*T. duloticus*	*T. pilagens*	*T. americanus*
hypothetical protein SINV_06866	Putative inorganic phosphate cotransporter	Putative inorganic phosphate cotransporter
hypothetical protein SINV_03497	Paired amphipathic helix protein Sin3a	Aminopeptidase N
Alpha-catulin	Zinc transporter ZIP1	RING finger protein 17
Thyrotropin-releasing hormone-degrading ectoenzyme	Uncharacterized protein	Sugar transporter ERD6-like 7
hypothetical protein SINV_12600	hypothetical protein SINV_09653	Matrix metalloproteinase-14
Pleckstrin-like protein domain-containing family M member 2	Kelch-like protein 10	hypothetical protein G5I_08549
F-box/LRR-repeat protein 20	Fatty acyl-CoA reductase 1	Receptor-type tyrosine-protein phosphatase beta
hypothetical protein SINV_03929	hypothetical protein EAI_01741	hypothetical protein SINV_02546
Major facilitator superfamily domain-containing protein 6		Cytochrome b5
Sphingomyelin phosphodiesterase		hypothetical protein SINV_09234
Zinc finger protein jing-like protein		hypothetical protein G5I_14818
Elongation of very long chain fatty acids protein		Putative ATP-dependent RNA helicase DDX23
Circadian clock-controlled protein		Trypsin-7
hypothetical protein SINV_04023		Circadian clock-controlled protein

The comparison of pathways associated with selected genes indicates that selected genes between species not only belong to different functional categories, but also to many different pathways (Additional file 5). In slavemakers, we identified 128 different pathways amongst the positively selected genes, the majority (77%) of which were also species-specific (χ^2^_1_ = 73.26, *p* < 0.001; Fig. 2c). The genes positively selected in hosts belong to 102 different pathways, the majority (70%) of which were species-specific (χ^2^_1_ = 29.82, p < 0.001; Fig. 2d). Nevertheless, more pathways were shared than expected by chance in hosts and also in slavemakers, except between the sister species *T. pilagens* and *T. duloticus* (Additional file 4: Table S2). The eight pathways shared among hosts are the metabolic pathway, biosynthesis of secondary metabolites, biosynthesis of antibiotics, p53 signalling, PI3K-Akt signalling, Wnt signalling, thyroid hormone signalling and the longevity regulating pathway. In slavemakers, four pathways were shared amongst all three species including metabolic pathways, biosynthesis of secondary metabolites, biosynthesis of antibiotics and endocytosis.

### Slavemaker-host pairs

**Fig. 3:**
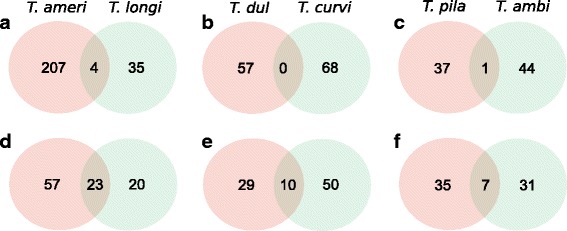
Venn diagrams depicting shared and “private” selected genes (**a**-**c**) and pathways (**d**-**f**) between host and slavemakers species pairs. For species name abbreviations please refer to Fig. 2

Genes under selection in slavemaker-host pairs should show little overlap due to their coevolution, as different traits are under selection in slavemakers and hosts. An exception could be cuticular hydrocarbon genes, when slavemakers try to mimic host profiles [77] and utilize the same genes as their closely related hosts. A more important cause of overlap in selected genes in slavemaker and host pairs might be, that both species inhabit the same habitat and therefore adapt to the same environmental conditions. We investigated the number and functions of positively selected genes and pathways between each slavemaker-host pair in order to make inferences on local adaptation. We found between none and four shared positively selected genes (Fig. 3a-c; Additional file 6), and 7–23 shared pathways between pairs (Fig. 3d-f). The number of shared genes and pathways was higher than expected in the pair including the most diverged parasite species *T. americanus – T. longispinosus*, and as expected by chance in the other two parasite-host pairs (Additional file 4: Table S3 and S4). Moreover, we tested whether slavemakers share more genes with their preferred host in contrast to the other host species. *T. duloticus* shares more genes with *T. longispinosus* (*n* = 5) in comparison to its preferred host *T. curvispinosus* (*n* = 0) (X^2^_1_ = 4.811, *p* = 0.028). In all other cases, the number of shared genes between preferred host and the other species did not differ (results not shown).

## Discussion

The slavemaker lifestyle evolved several times independently in ants, with a hotspot of slavery evolution in the genus *Temnothorax* [37]. As slavemakers and their hosts are engaged in a constant co-evolutionary arms-race, the evolution of ant slavery is tightly linked to the evolution of behaviour, physiology and morphology in their hosts [78–80]. The focus of our study was the genomic basis of the (co-) evolution of the slavemaker and host lifestyle. We were thus interested in identifying genes with signatures of positive selection in slavemakers and hosts respectively. Furthermore, we asked whether or not, and at which organisational level, the three slavemaker/host species show signs of genetic convergence; or whether each species follows its own specific evolutionary trajectory.

### Positively selected genes

We identified twice as many genes under positive selection on branches leading to slavemakers as compared to host branches. This finding is in line with our expectation that the derived slavemaker mode of life should have led to the selection of more genes in comparison to the ancestral host lifestyle. However, based on the number of species-specific selected genes it becomes evident that 70% of the positively selected genes in slavemakers can be assigned to *T. americanus* only. All other slavemaker and host species have comparable and lower numbers of positively selected genes. *T. americanus* is the most distantly related species in this taxon and its behaviour and morphology are most derived from the other species. *T. americanus* workers have large square heads, which make them easily distinguishable from their hosts. They do not engage in normal worker behaviour, such as brood care or foraging, and are so dependent upon enslaved hosts that *T. americanus* will starve to death if not fed. During raids, they manipulate host behaviour via the release of glandular secretions [11, 41, 46], but never use their stinger, which is a typical behaviour for other *Temnothorax* hosts during aggressive interactions [7, 41, 48]. Foraging and brood care are standard behavioural repertoires in the hosts, and in the lack of slaves, will still be performed by *T. duloticus* [41, 44] and *T. pilagens* (pers. observation) slavemakers to some extent. In addition to many lifestyle differences, the longer evolutionary history with the possibility for co- and counter adaptations to *T. longispinosus* and its host ancestor, might explain the large number of positively selected genes in *T. americanus* in contrast to the other species.

Within the 309 positively selected genes on the branches to slavemakers, we were able to identify several candidate genes with a possible link to their slavemaker lifestyle. Amongst these, three different DNAJ-like protein subfamily members, which are heat shock protein homologs, and function as co-chaperons. They are involved in stress response in humans [81], and could thus play a role during stressful slave raids into fiercely defending host colonies.

*Tachykinin* is positively selected on the branches leading to hosts and might thus be a candidate gene for the host lifestyle. *Tachykinin* has been linked to aggressive behaviour in *Drosophila* [82 and authors therein], and recently also in ants [83]. *Temnothorax* hosts need to defend their nest aggressively, not only against intra-specific intruders, but particularly against slavemakers. Thus selection might specifically act on this gene in hosts. This is further corroborated by the fact that intra-specific aggression increases with the prevalence of the slavemaker *T. americanus* in the population [54].

In comparison to an accompanying gene expression analysis including the same six host and slavemaker sequence data [64], we found six genes to be both, differentially expressed and under positive selection in hosts and 36 in slavemakers. These genes are thus prime candidates for the evolution of the slavemaker and host lifestyle. Firstly, they are directly involved in raiding behaviour. Secondly, they show signs of positive selection. Among these genes *Trypsin-7* was identified in two host species and the slavemaker *T. americanus*. *Trypsin-7* is known for its function in digestion, e.g. it is blood meal induced in *Anopheles gambiae*, and may also play a role in host seeking behaviour [84]. It may thus be involved in host seeking behaviour in slavemakers and slavemaker detection in hosts. Endogenous daily (circadian) and annual (circannual) rhythms serve as biological clocks that provide the major basis for timing in most organisms [85]. Annual timing mechanisms regulate seasonal timing of reproduction, moult, and hibernation [86 and authors therein]. Positive selection on the *circadian clock controlled protein*, in slavemakers compared to hosts, suggests that this gene may regulate the aberrant activity patterns of slavemaker workers. Slavemakers are only active during raiding season in summer, and are taken care of for the rest of the year by the slaves [39, 87]. This changed activity pattern might thus be manifested by changes in the circadian rhythm. Two more genes of interest with possible direct link to the slavemaker evolution are “*Elongation of very long chain fatty acids protein*” in *T. duloticus* and *Fatty acyl-CoA reductase 1* in *T. pilagens*. Both genes could be involved in the synthesis of cuticular hydrocarbons and thus might play a role in the avoidance of host recognition [88]. Indeed, a recent study on the cuticular hydrocarbon profiles of the same six species, revealed that slavemakers show consistently different chemical profiles than the three host species [89]. A recent switch to a parasitic lifestyle could thus have led to selection on genes underlying hydrocarbon synthesis.

In slavemakers only four pathways are shared among species. Three of these (metabolic pathways, biosynthesis of secondary metabolites, and biosynthesis of antibiotics) were also identified in the hosts, and only endocytosis is slavemaker specific. In hosts, the genes with signatures of positive selection belong to 102 different pathways, eight of which are shared among the three species. The PI3K-Akt pathway is part of the mTOR pathway regulating the cell cycle, and also known for its function in longevity [90]. Furthermore the longevity regulating pathway is shared among all three host species, reinforcing the importance of longevity within these host species. Despite their small body and colony size *Temnothorax* ants are quite long-lived with workers living up to a few years and queens over two decades [91]. Social Hymenopterans are known for a change in the longevity-fecundity trade-off with queens being both long-lived and highly fecund compared to the short-lived sterile workers [92–97]. We identified two pathways, which may play a role in morphological differences between the species and their adaptive divergence. The Wnt signalling pathway is known for its role in regulating key events during embryonic patterning and morphogenesis [98], and the thyroid hormone signalling pathway (in humans) is involved in the regulation of growth development and metabolism. The latter has been shown to play a role in the adaptive divergence of sticklebacks [99].

### Slavemaker – Host pairs

Besides determining similarities in possible selection pressures within slavemakers and within hosts, we additionally investigated similarities in slavemaker-host pairs, because of their shared environment. We hypothesized that genes shared by both slavemakers and hosts with signatures of positive selection might give indication on local environmental selection pressures; though they could also represent genes involved in the co-evolutionary arms race. However, on the gene level there is hardly any congruence between slavemaker and host pairs (0–4 overlapping genes). On the pathway level, some of the above mentioned candidates appear, such as thyroid hormone signalling pathway in *T. ambiguus* and *T. pilagens*, PI3K-Akt and Wnt signalling pathway between *T. americanus* and *T. longispinosus*. In the *T. duloticus – T. curvispinosus* pair we identified circadian rhythm as well as the FoxO signalling pathway. Among others the latter coordinates the response to environmental changes, including metabolic stress (starvation) and oxidative stress [100]. Hence, these two pathways may give evidence for environmental selection pressures, e.g. temperature, seasonality, or food availability, experienced by *T. duloticus* and *T. curvispinosus* which co-occur in the same environment, in comparison to the other four species which are from different locales.

## Conclusions

Our positively selected gene analyses revealed several candidate genes with a possible link to the slavemaker lifestyle, which are involved in the cuticular hydrocarbon profile composition, thus species recognition, and the aberrant activity pattern of slavemaker workers. To verify the functional and phenotypic importance of these candidates will now be the next step.

Furthermore, the results show little overlap of selected genes between species. On the pathway level however, we find higher congruence between species than expected, even though the majority of selected pathways remain species specific. Furthermore, the genes under positive selection belong to a wide variety of functions, as indicated by negative results in the enrichment analyses. The same pattern was identified in social parasitic cape honeybees [37]. It thus seems that the evolution of social parasites, including slavemakers is a broad encompassing process with species-specific evolutionary trajectories, based on selection in many genes with different functionality and pathway affiliation. Our results support the hypothesis that evolution is the unrepeatable result of stochastic events with highly contingent effects [101].

## Additional files

Additional file 1: Information on sample collection sites and year for each species. (DOCX 112 kb)

Additional file 2: Summary of read counts and contig information per species. (DOCX 14 kb)

Additional file 3: Codeml results on selected gene identified on branches leading to slavemakers and hosts, as well as each single species. Plus information on shared and private selected genes. (XLSX 135 kb)

Additional file 4: Results of randomisation statistics. (DOCX 16 kb)

Additional file 5: KEGG pathways assigned to positively selected genes per branch. (XLSX 21 kb)

Additional file 6: Summary shared selected genes between slavemaker-host species pairs. (XLSX 39 kb)

## Abbreviations

L:D: Light: dark
dN/dS: Ratio of non-synonymous to synonymous mutations
mtDNA: Mitochondrial DNA
