# Supplementary figures and images for: Species-specific genes under selection characterize the co-evolution of slavemaker and host lifestyles
Source: BMC Evol Biol. 2017 Dec 4;17:237. doi: 10.1186/s12862-017-1078-9 (PMC5715652; doi:10.1186/s12862-017-1078-9)

Supporting Information S1: Information on sample collection sites and year for each species.

**
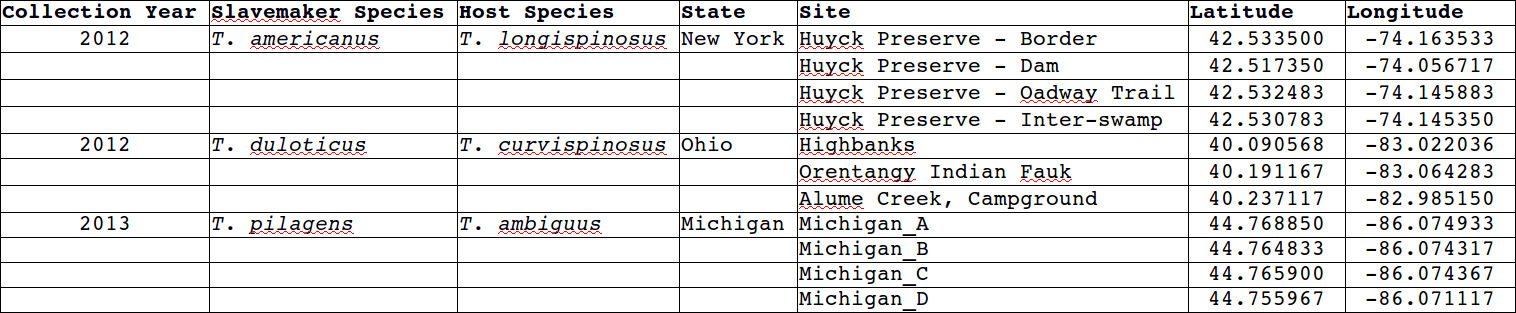
**

Supplement: Supplementary file 1 — Information on sample collection sites and year for each species. (DOCX 112 kb) [file 12862_2017_1078_MOESM1_ESM.docx]
