# Supplementary material for: Species-specific genes under selection characterize the co-evolution of slavemaker and host lifestyles
Source: BMC Evol Biol. 2017 Dec 4;17:237. doi: 10.1186/s12862-017-1078-9 (PMC5715652; doi:10.1186/s12862-017-1078-9)
Supplement: Supplementary file 2 — Summary of read counts and contig information per species. (DOCX 14 kb) [file 12862_2017_1078_MOESM2_ESM.docx]

Supporting Information S2: Summary of read counts and contig information per species

|  | Raw Reads (bp) | After Trim (bp) | # Contigs | ø Len. (bp) | Blast Hits |
| --- | --- | --- | --- | --- | --- |
| *T. americanus* | 172,532,840 | 166,942,700 | 54,054 | 1,351 | 11,809 |
| *T. ambiguus* | 125,458,806 | 116,315,281 | 43,664 | 1,443 | 11,350 |
| *T. curvispinosus* | 272,428,981 | 259,764,589 | 79,277 | 1,214 | 18,396 |
| *T. duloticus* | 244,567,909 | 233,235,994 | 57,994 | 1,345 | 10,407 |
| *T. longispinosus* | 198,594,137 | 191,964,863 | 56,122 | 1,347 | 12,240 |
| *T. pilagens* | 150,162,599 | 140,569,260 | 48,783 | 1,341 | 10,206 |
