# Supplementary material for: Species-specific genes under selection characterize the co-evolution of slavemaker and host lifestyles
Source: BMC Evol Biol. 2017 Dec 4;17:237. doi: 10.1186/s12862-017-1078-9 (PMC5715652; doi:10.1186/s12862-017-1078-9)
Supplement: Supplementary file 4 — Results of randomisation statistics. (DOCX 16 kb) [file 12862_2017_1078_MOESM4_ESM.docx]

**Supplement Tables Shared Stats**

For the following tables please note that species names are abbreviated: *Temnothorax longispinosus* = longi*, T. curvispinosus* = curvi*, T. ambiguus* = ambi*, T. americanus* = ameri*, T. duloticus* = dul*, T. pilagens* = pila.

A randomisation procedure implemented in a custom Python script was used to assess whether observed intersections among pairs of species would be expected by chance. We used 10,000 replicates to infer how often an observed intersection of size i of x and y positive draws from a base population of size z was larger or smaller than those from random draws.

**Codeml**

Table A: Results of the randomization test statistics on shared positively selected genes between species pairs of hosts and slavemakers.

| Comparison | Random expectation | Observed | P (↑more, ↓less than expected) |
| --- | --- | --- | --- |
| longi-curvi | 12 | 0 | <0,001 (↓) |
| longi-ambi | 8 | 1 | <0,001 (↓) |
| ambi-curvi | 13 | 2 | <0,001 (↓) |
| pila-americ | 17 | 3 | <0,001 (↓) |
| americ-dul | 25 | 3 | <0,001 (↓) |
| pila-dul | 5 | 2 | <0,001 (↓) |

Table B: Results of the randomization test statistics on shared pathways based on positively selected genes between species pairs of hosts and slavemakers.

| Comparison | Random expectation | Observed | P (↑more, ↓less than expected) |
| --- | --- | --- | --- |
| longi-curvi | 8 | 14 | 0,005 (↑) |
| longi-ambi | 5 | 16 | <0,001 (↑) |
| ambi-curvi | 7 | 17 | <0,001 (↑) |
| pila-americ | 9 | 16 | 0,003 (↑) |
| americ-dul | 8 | 16 | 0,0012 (↑) |
| pila-dul | 5 | 5 | ns |

**Comparison slavemaker-host pairs**

**Codeml**

Table E: Results of the randomization test statistics on shared genes under positive selection between slavemaker and host species pairs.

| Comparison | Random expectation | Observed | P (↑more, ↓less than expected) |
| --- | --- | --- | --- |
| americ-longi | 1 | 4 | 0,009 (↑) |
| dul-curvi | 0,7 | 0 | ns |
| pila-ambi | 0,2 | 1 | ns |

Table F: Results of the randomization test statistics on shared pathways of genes under positive selection between slavemaker and host species pairs.

| Comparison | Random expectation | Observed | P (↑more, ↓less than expected) |
| --- | --- | --- | --- |
| americ-longi | 10 | 23 | <0,001 (↑) |
| dul-curvi | 7 | 10 | ns |
| pila-ambi | 5 | 7 | ns |
